# Person-centred interventions for problem gaming: a stepped care approach

**DOI:** 10.1186/s12889-021-10749-1

**Published:** 2021-05-06

**Authors:** Jennifer J. Park, Laura Wilkinson-Meyers, Daniel L. King, Simone N. Rodda

**Affiliations:** 1 School of Population Health, Faculty of Medical and Health Sciences, University of Auckland, Private Bag 92019, Auckland, 1142 New Zealand; 2 College of Education, Psychology, & Social Work, Flinders University, GPO Box 2100, Adelaide, South Australia 5001 Australia

**Keywords:** Gaming disorder, Treatment, Internet gaming, Screening, Intervention

## Abstract

**Background:**

Problem gaming is reported by approximately 1–3% of the population and is associated with decreased health and wellbeing. Research on optimal health responses to problem gaming remains limited. This study aimed to identify and describe the key components of a person-centred approach to interventions for problem gaming for individuals who voluntary seek assistance.

**Methods:**

Online interviews were conducted with 20 adults (90% male; M_age_ = 23y) currently seeking help for problem gaming. The interview protocol was guided by a health care access framework which investigated participants’ experiences and needs related to accessing professional support. Transcripts were analysed in NVivo using qualitative content analysis to systematically classify participant data into the themes informed by this framework.

**Results:**

Participants had mixed views on how the negative consequences of problem gaming could be best addressed. Some indicated problems could be addressed through self-help resources whereas others suggested in-person treatment with a health professional who had expertise in gaming. Participants described the essential components of an effective health service for problem gaming as including: valid and reliable screening tools; practitioners with specialist knowledge of gaming; and access to a multimodal system of intervention, including self-help, internet and in-person options that allow gamers to easily transition between types and intensity of support.

**Conclusion:**

A comprehensive health care approach for interventions for problem gaming is in its infancy, with numerous service access and delivery issues still to be resolved. This study highlights the importance of involving individuals with gaming-related problems in developing solutions that are fit for purpose and address the spectrum of individual preferences and needs. These findings recommend a stepped healthcare system that adheres to evidence-based practice tailored to each individual and the implementation of standard assessment and routine outcome monitoring.

## Background

Problem gaming is reported by approximately 1–3% of people internationally [1–4]. People with gaming problems report issues including reduced quality of physical health (e.g., sleep disruption), psychological well-being (e.g., anxiety and depression), social life (e.g., impaired quality of relationships) and legacy problems such as reduced employment or educational attainment [5–7]. People with gaming problems also report other mental health conditions including anxiety, depression, ADHD, social phobia, and anxiety. Problem gaming appears to be more prevalent in male gamers [8, 9]. Following a provisional status for ‘internet gaming disorder’ in the DSM-5 (American Psychiatric Association, 2013), “Gaming Disorder” was officially adopted at the World Health Assembly in May 2019 as a diagnosis in the eleventh edition of the International Classification of Diseases (ICD-11) [10]. Gaming disorder within the ICD-11 is characterized by increasing priority given to gaming over other activities, impaired control over gaming, and functional impairment due to gaming for a period of at least 12 months in most instances. Recognition in the ICD-11 is an important step toward advancing knowledge and standardising approaches to the condition. However, the concept of problem gaming as an addictive disorder is the topic of extensive debate [2, 11–19]. For instance, measurement inconsistencies add to problems identified with specific criteria listed in the IGD classification, particularly the relatively low specificity of the tolerance and mood modification criteria [20]. In the current paper we refer to gaming problems as inclusive of Gaming Disorder.

Recent systematic reviews and meta-analyses have examined the quality and effectiveness of prevention of gaming problems [21–24] and treatment [25–28]. To date, research on prevention has tended to focus on school-based prevention programs. Much of the work in this area has been conducted in Asian settings [29, 30], including South Korea and China, where there have been parallel developments in trialling targeted technology-restriction measures including content filters and gaming time limits aimed particularly at younger users [31–34]. In terms of treatment, the most common approach has been cognitive-behavioural therapy (CBT), usually delivered in brief individual and group-based formats, and other non-CBT psychotherapeutic interventions [25–27, 35, 36]. CBT for gaming problems may be an effective short-term approach for reducing gaming problems and depressive symptoms, but more studies with follow-up are needed to assess longer-term gains [35, 37]. Pharmacological interventions have predominantly employed antidepressants (i.e., bupropion and escitalopram), but their effectiveness is currently unclear due to lack of controlled trials [14]. Some treatment centres provide brief voluntary retreats from digital technologies, group therapy and social activities, but these options may be financially burdensome and have limited evidence for their long-term efficacy.

The growing body of literature examining early intervention and treatment for gaming problems highlights several important gaps [25, 26, 30, 36]. Zajac et al.’s [26] review of 15 studies reported that the majority had targeted school age samples with just six studies involving adults. The majority of participants were male and almost all were conducted in university settings, however there was some heterogeneity in terms of problem severity (mild, moderate and severe problems) and time spent gaming (ranging daily to weekly sessions, and very low to high levels of gaming time). Treatment was often delivered over 4 to 8 weeks, and almost all were delivered via in-person consultation. Other reviews [25, 36] have reported most studies have been conducted in East Asian settings, indicating a need for cross-cultural perspectives. There are well-documented problems of validity across a wide range of assessment tools, including those employed in intervention studies [38, 39]. A related problem is that many intervention studies, particularly those in Asia, have relied on problematic internet use measures to evaluate gaming. Together, these reviews suggest that interventions may be enhanced by a range of improvements and wider consideration of alternative treatment approaches and delivery methods.

Very few people experiencing gaming-related problems will access the healthcare system [24, 40]. A longitudinal study involving over 4000 adults in Canada reported just 4.5% of people with gaming problems (*n* = 69) had sought professional help [41]. The type of help surveyed included a family physician, psychologist, psychiatrist, counseling service or telephone helpline. Rates of professional treatment-seeking for other addictive behaviours is approximately 10% [42], much higher than is reported for gaming problems. The reason for the low rate of treatment seeking may be due to structural issues such as the homogeneity of available treatments and lack of available options. Most research has investigated intensive and in-person treatments which may not be the right kind of support for people without other complex or co-occurring issues. For those that could benefit from in-person treatment, it may be that individual barriers such as procrastination, impulsivity, shyness or introversion impede help-seeking [43–46]. Another possibility is that some individuals with gaming-related problems seek out information, social support and assistance from less formal, convenient sources (e.g., online support groups), and this is sufficient to address the problem [47].

People with gaming problems report co-occurring mental health issues which may be a precursor or concurrent to gaming problems [48]. High rates of depression, anxiety, substance use and gambling disorder may mean people with gaming problems attend other services for more acute issues and therefore do not report seeking help for gaming [46]. Lau et al. [49] for example, examined the records of 5820 clinically referred youth in the Canadian mental health system and reported that moderate to severe problematic gaming was reported by 13% of the sample; however, most of the sample had been referred for issues including threat or danger to self or others, or other psychiatric symptoms. This suggests a need to explore how early intervention and treatment for gaming problems can be provided by a range of different health care providers.

Taken together, the evidence suggests a need to broaden the scope of research into interventions so as to provide a stepped care approach to early intervention and treatment. The current study interviewed people experiencing gaming problems to gather their views of the components of an effective integrated health care approach. Although the prevalence of gaming problems in New Zealand (NZ) is currently unclear, there is evidence of problematic gamers in this region [50] and surveys of psychiatrists and mental health professionals that suggest NZ gamers and their support networks may seek help via addiction and related services [51, 52]. The aims of this study were to: (i) describe the experiences and needs of people seeking help for gaming problems; and (ii) identify the optimal components of a health care system to support early intervention and treatment of gaming problems.

## Methods

### Participants

Participants were drawn from a larger study examining the impact of a brief online intervention for gaming problems [53]. The larger study involved 50 gamers who reported an intention to limit or reduce their gaming in the next 30 days. Participants were also required to be aged 18 years or older and not currently seeking in-person treatment. The current study involved sequential recruitment of the first 20 participants willing to engage in a semi-structured online interview examining the needs, experiences, and preferences for treatment-seeking in New Zealand. Recruitment occurred over a one-month period between April and May 2019 through social media advertising, posters in the Auckland community (university, schools, supermarkets) and word of mouth. While there was no specific remuneration for the current study, all participants received a NZD$50 shopping voucher for completing follow-up evaluation as part of the main study. The research was approved by the University of Auckland Human Participants Ethics Committee (022614).

Table 1 presents a summary of participant information. The majority of participants were male (*n* = 18, 90%) and the average age of participants was 23 years (range: 20 to 35 years). The sample primarily identified as New Zealand European (*n* = 10, 50%) and as Asian (*n* = 7, 35%). Using the Gaming Addiction Scale-21 [54] to screen for problematic gaming, 16 participants were classified as problematic gamers (endorsed four or more of seven item areas). Four participants indicated concerns about their gaming but did not meet the GAS cut-off score. The average hours spent online per week was 29 (SD = 23) with an average frequency of 9 sessions per week (SD = 4.7).

**Table 1:** Participant characteristics at baseline

ID	Age range	Sex	Employment	GAS criteria	Frequency gaming p/w	Hours gaming p/w	Psychological Distress (K6)
1	20–24	Female	Student (not employed)	Unmet	17	6	24
2	20–24	Male	Unemployed, not looking for work	Problem gaming	11	21	12
3	25–29	Male	Employed part time	Problem gaming	18	70	21
4	20–24	Male	Student (not employed)	Problem gaming	9	74	22
5	20–24	Male	Student (not employed)	Gaming Disorder	11	40	15
6	35–39	Male	Other (not stated)	Gaming Disorder	7	74	7
7	20–24	Male	Student (not employed)	Problem gaming	5	10	19
8	20–24	Male	Employed part time	Gaming Disorder	7	47	19
9	20–24	Male	Unemployed, looking for part time work	Problem gaming	10	34	18
10	20–24	Male	Employed part time	Gaming Disorder	10	18	18
11	20–24	Male	Employed full time	Problem gaming	18	28	21
12	20–24	Male	Student (not employed)	Problem gaming	4	4	17
13	20–24	Male	Unemployed, not looking for work	Problem gaming	9	17	14
14	20–24	Female	Employed part time	Unmet	3	5	10
15	20–24	Male	Employed part time	Unmet	7	14	13
16	25–29	Male	Student (not employed)	Problem gaming	7	13	21
17	20–24	Male	Unemployed, looking for part time work	Gaming Disorder	16	58	14
18	20–24	Male	Employed full time	Problem gaming	11	25	17
19	20–24	Male	Employed part time	Unmet	2	10	11
20	30–34	Male	Employed full time	Problem gaming	7	10	23

*GAS* Game Addiction Scale, *K6* Kessler-6

### Procedure

All participants completed a participant information sheet and informed consent form in Qualtrics survey software. This included completion of demographic (age, sex, employment, ethnicity) and gaming-related measures. The survey included the 21-item Game Addiction Scale [54] which measures seven dimensions of problem gaming (i.e., salience, tolerance, mood modification, relapse, withdrawal, conflict and problems). Participants also completed the Time Line Follow Back [55, 56] to determine the duration and frequency of gaming, and the Kessler 6 measure of non-specific psychological distress [57].

**Fig. 1:**
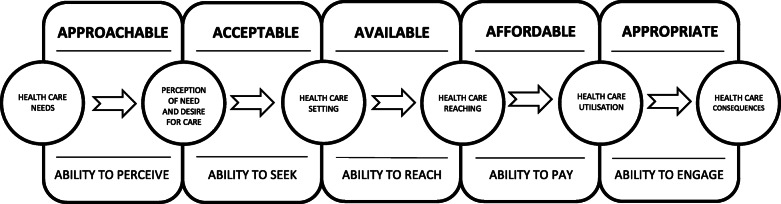
Conceptual framework of access to health care based on Levesque et al. [58]

A semi-structured interview schedule was developed based on the components of the person-centred model for accessing health care by Levesque (2013) and colleagues [58]. As indicated in Fig. 1, this access framework highlights the important relationship between the design of health services and the capabilities of those who seek help to facilitate access to appropriate health care and improved health.

The purpose of semi-structured interviews was to identify needs and preferences for support and treatment, specific to gaming problems. The first part of the interview schedule focused on the capabilities of gamers, and the individual barriers and facilitators to help seeking. This included the questions: *“I understand that you are interested in doing something about your gaming. What made you make this decision?”* and “*What might stop you from getting help? What could help address these barriers?”* Additional prompts were included in the semi-structured interview schedule to continue the conversation if it became stalled (i.e., the participant needed more context on the nature of the questions). These included: reasons for wanting to change gaming and reasons for seeking help; barriers and facilitators to help seeking; and knowledge of services. The second part of the interview schedule focused on support and service needs. Specifically, participants were asked: “*We are looking at services that could be developed for people wanting help for gaming. Let’s say you decide you want to seek help in the future. What would be important to you in a service?*” Prompts included accessibility (cost, waiting times, travel, location, type of support), quality (treatment types, qualifications, relationships, desired resources) and equity (considerations for culture, age, sex and social disadvantage).

Interviews were conducted by a postgraduate population health student under supervision. The interviewer was trained in motivational interviewing and the administration of a semi-structured interview schedule. Interviews were by appointment and delivered via the participants’ preferred instant messaging platform (Facebook Messenger, Skype, WhatsApp). Instant messaging was used instead of recording video call to provide greater participant anonymity, increase personal disclosure, and provide more coherent verbal expression for qualitative analysis. Transcripts were created by copying and pasting the online conversation into a word document. The average time per interview was 90 min (range 40 to 180 min) and the average word count was 839 (range 377 to 1988).

### Data analysis and application of framework

We applied the conceptual framework of person-centred access to health care to understand how services can meet the needs of people with gaming problems [58]. This framework systematically describes the client and provider factors necessary to facilitate the entire client experience from the point of identifying a health care need to the ultimate achievement of improved health outcomes (see Fig. 1). From the client perspective, the framework outlines five processes that influence access to support and treatment. These include the *ability to perceive* a need for treatment (including health literacy, health beliefs and knowledge of services); *ability to seek*, *reach* and *pay* for treatment as well as an *ability to engage* (facilitating minimal attrition and maximum adherence). From the treatment provider perspective, client access to health care is influenced by their *approachability* (service information, screening tools), *acceptability* (values and culture of the organisation and support for client autonomy), *availability* (physical location, mode of delivery, qualifications of provider and flexible opening hours), *affordability* (time and resource costs) and *appropriateness* (delivery of evidence-based treatment tailored to client need). A qualitative content analysis [59] was used to group our data into this framework. This method was selected because we wanted to systematically classify participant data into themes guided by an established framework [59].

NVivo software was used to assist the qualitative content analysis. Transcripts were read twice for familiarity with the data and initial codes developed. Two complete transcripts were coded by two researchers (JP and SR). The codes were discussed and disagreements were resolved through consensus. Common codes were grouped by the first author into themes that were adjusted to accommodate additional data. As outlined by Hsieh and Shannon [59], this data was then grouped into broad categories which reflected the selected framework. Data that could not be coded into one of the broad categories were re-examined to describe different manifestations of treatment needs and these were merged into the framework and noted accordingly. The findings were structured according to: (1) participant ability to *perceive, seek, reach, pay* and *engage* with health care, which is supported with illustrative quotes, and (2) the proposed components of a health care system response specific to gaming problems. We reported the proportion of participant statements (*n* = 568 statements that were coded) that align with each of the five components of the framework. All 20 participants provided at least one statement that was relevant to the framework. Where quotes were used, these were de-identified to ensure participant anonymity. Quotes were also cleaned to improve readability (i.e., spelling and punctuation) as well as clarity (i.e., grammar corrected).

## Results

### Treatment needs and preferences

Table 2 presents a summary of the five components of the framework from the client perspective. The most frequently discussed participant experiences related to *ability to seek* help with relatively less discussion on ability to pay for services. The *ability to perceive* a need for help was focused on health beliefs and health literacy (10% of statements from the 20 participants). Health beliefs focused on the seriousness of gaming problems in relation to the amount of harm. Participants indicated they had a reasonable understanding of the characteristics of gaming problems including its nature, prevalence, and resultant harms. As indicated in Table 1, participant experiences ranged from gaming being a problem that could be managed, through to a range of negative consequences including reduced quality of health, work, sleep and social life. Participants reported that family and friends were a barrier to perceiving a problem in that gaming problems were not real, trivial, or a “soft” addiction. Participants stated this increased their feeling of being stigmatised and a key reason to delayed help-seeking. There were many different reported reasons for deciding to change as indicated by the cons of gaming starting to outweigh the pros. For some participants, there were co-occurring issues such as alcohol, cannabis or mental health issues that were more acute and/or deserving of attention.

**Table 2:** Mapping participant experiences against the five constructs

Construct	Participant experiences	Indicative quotes
Ability to perceive a need for support	**Health beliefs** • Perceived harm included wasted time, energy, ability to focus on more meaningful achievement, entrenched lifestyle, ignoring personal hygiene or disrupted sleep. • Decisional balance involved the benefits of gaming (positive hobby, social connection, skill building, enjoyable, relaxing) versus negatives (health, work, social). Cons of gaming start to outweigh the benefits. • The reasons for excessive gaming included loneliness, isolation, boredom, lack of excitement, feelings of safety, respect (player), sense of achievement, relaxation, distraction, altering mood such as to feel good, fill a void, manage stress. • Other mental health (depression or anxiety) or substance use disorders (alcohol, cannabis) were perceived as more acute and associated with immediate harm. **Health literacy** • Understand the potential seriousness of addiction in terms of the risk for harm. This includes understanding the nature of addiction and how it relates to gaming. • Understand the prevalence, biological and psychological characteristics, harm and risk factors associated with problematic gaming.	• *Gaming addictions certainly appear to be seen as “soft” addictions, especially in comparison to chemical addictions, and as such are not taken seriously until it’s too late.* (#2) • *Gaming has become a way of life for me recently to the point of gaming from when I wake up till when I sleep.* (#6) • *I think that the addiction just isn’t seen as being as serious despite the hours and money spent on it.* (#10) • *I think gaming addiction in particular is sometimes regarded as a bit more trivial or not as much of a problem as addictions. I’ve tried talking about it with a few people I am close with and it’s kind of been brushed off, and I haven’t really felt comfortable going to a counselor for it either. (#5)* • *I think it works like any other addiction. Whether physical or mental we build a reliance on things. Mine is gaming for dopamine I guess.* (#18) • *My addiction feels like it’s taking a big chunk of my ability to actually think of things I want to accomplish by overcoming it. I am near failing at university, I can’t get by on just been bright anymore. Actually, need to do hard work, which sadly is difficult to start. (#3)* • *When I am feeling a bit down, I’ll tell myself just to chill out and play something for a bit, but it kinda spirals from there. (#10)* • *The amount of time I spend gaming has a much bigger negative impact on my life (health, work, sleep, social) than positive. (#5)*
Ability to seek support	**Knowledge of services** • Limited knowledge of where to seek help. Limited knowledge of how treatment works. **Appropriateness of help seeking** • A perception that services already have too much need and that gaming is not as important or harmful as other issues. Associated view that in-person services are unable or unwilling to make time for gaming issues. • Locating gaming treatment with perceived more serious issues discourages help-seeking. • Services do not convey a knowledge of gaming culture or specific approaches to treatment. • Friends, partners, and family provided encouragement to game less, but limited encouragement, support or motivation to seek help or engage in behaviour change. **Stigma and shame** • A feeling of shame or embarrassment that help is needed and that the issue is beyond self-management. • Help seeking is not typical amongst gamers and the gaming culture does not support leaving gaming networks. **Age and sex appropriate services** • Perception that treatment may not be age or sex appropriate. This includes the way information is currently provided (needs to be brief and targeted) as well as the type of information conveyed (e.g., relevant to young adults). • Recognise that females have gaming problems too and likely experience additional stigma. **Desire for autonomy** • A desire to choose personal gaming reduction goals (stay the same, reduce, abstain) and to determine the speed of change. • A preference to avoid treatment that does not respect individual choice. Interest in self-management and a do-it-yourself approach. • Preference to be treated as an adult and be able to make own decisions.	• *I don’t know where to seek help for a potential gaming addiction. I don’t know of any available services for specifically this.* (#2) • *I think if it was made known that these other programs could also assist with things such as a gaming habit it would help people like me take the issue more seriously because until recently it never seemed a serious enough issue next to the other problems these programs deal with.* (#7) • *People I know have told me that I should “solve my problem” and that “I need to stop gaming” but they technically have never actually encouraged me to say seek a service to assist in the matter*. (#5) • *I would be reluctant to engage in counselling because of the strain on local services and cost of private services. (#2)* • *Other programs dedicated to helping people overcome personal issues tend to be focused on more serious problems such as mental health, living conditions, or family issues, and therefore these programs don’t advertise the fact that they could help with something like this.* (#7) • *Yeah of course there’s stigma around it. It’s quite embarrassing, especially today’s university students since my friends play the same amount as I do it isn’t embarrassing within our circle however outside of that people will always question and shame it.* (#8) • *I feel like people would perceive me in a strange/snowflake way if I were to get help for ‘playing too many games’.* (#5) • *Anyone my age probably wouldn’t want the information to be incredibly detailed and long but more key phrased and engaging.* (#18) • *Not being treated like a child when I’m not is a big thing though. If they don’t respect me what’s the point? The service did not understand addictive behaviour, only physical addiction, and didn’t have an understanding of the factors that relate to addiction enough to actually help and I ended up giving up seeking help.* (#4)
Ability to reach for support	**Transport, travel and wait times** • Few service options mean travelling distances to receive help. Ideally it is available within own town or region. • Willing to travel around 30 min to receive treatment. Must have access with public transport. • Willing to wait for an appointment because it is not generally acute or life threatening. Acceptable wait times ranged from a few days to a few months. **Location** • Co-location with addiction services or problem gambling. Could be co-located with a university counselling service. • Access to advice and screening from primary care providers. Facilitate opportunistic interventions. • Available weekends and in the evenings. • If online there was a preference that the person had some local context. **Modality** • Multiple modalities including in-person and online. Need for flexible, convenient and discrete access as required. Online or face-to-face individual or group treatment. • Smartphone access for tracking behaviour and getting help as needed, and a website for screening and information.	• *It should be accessible. Have multi-platform ideally, with in-person and online communication pathways.* (#2) • *I reckon confidentiality is very important. And I prefer talking over messaging services rather than face-to-face. (#4)* • *A face-to-face group will probably be my optimal one as you can also share the issue with others which I think helps the success of dealing with an issue. (#14)* • *I’d much rather email or text someone to set up a meeting or begin a program than have to call or come in to somewhere to do this.* (#7*)* • *I like online services more than text/phone because I’m more used to typing online, it allows me to think through my words as I’m speaking for clear communication and reading replies is easier than listening to them. Online communication is great when you can’t make it into town easily.* (#2) • *I think maybe an online session chat is the best. Most people who are addicted to video games are shy, introverted* etc. *it is hard for them to actually come out and seek help.* (#16) • *If it was closer to exam time, I’d be a bit more desperate and therefore want help as quickly as possible, but other times through more relaxed parts of the year I’d be willing to wait a week or two.* (#7)
Ability to pay for support	**Direct fees** • Expectation that there would be a cost to attend in-person treatment. The recommended tiered approach based on income. For students the reasonable amount per session was around $20 and for those who were employed around $100. • Expectation that there could be private and public options and that health insurance would cover the fees. **Factors impacting ability to pay** • Expected value for money. Willing to pay more for better and more effective treatment. Likely only want to pay if there were serious harm or impact. • Difficulty getting time to attend appointments due to inflexible work or study. • Transition away from family home to university or community living put pressure on finances. Money was spent on gaming which decreased the ability to spend money on treatment.	• *I am a student and currently not working, so I cannot afford the service if it’s over $20. The cheaper the better.* (#1) • *Given that general counselling sessions normally range to about $100 I could probably accept say 150–200 given expertise, specialisation and success rates.* (#3) • *Depending on how long the sessions are, I would say $20 an hour, because if I can take the advice from the counsellor and use it, it would be worth it and it’s a reasonable price.* (#14) • *People most advantaged would be people with high incomes that could be more flexible with taking time off as well as affording the service itself.* (#10)
Ability to engage with support	**Service delivery characteristics** • Confidential, private and capacity for anonymity. Culturally appropriate. • Pre-treatment screening so as to not waste time. • Multiple modalities (face-to-face, group, online) and blended treatment (different types of service options such as peer and one-to-one). Single session or ongoing treatment. • Peer support forums moderated by a professional. • Able to be tailored to need (varying intensity from screening and drop-in to residential care). • Resources include online and written materials. **Treatment needs** • Skills development for relationships and social situations, emotional intelligence, time and stress management and sleep hygiene. • Treat underlying comorbidities such as impulsivity, substance use (alcohol, cannabis), depression and anxiety. • Enhance self-efficacy and belief in capacity to change. Support increased accountability, commitment, goal setting, urge management and self-monitoring. **Quality of providers** • Professional but not formal with demonstrated unconditional positive regard. Facilitate empathic and approachable partnerships. • The provider should understand gaming culture and technology and be an expert in treatment of gaming. **Adequacy of service** • Participants should be satisfied with the quality of service and experience the treatment as effective.	• *Some pre-involvement lead-up (such as questions like the ones we talked about earlier) so that the professional I was seeing could jump straight into tackling the problem from the first time I met them.* (#3) • *For me I feel like confidentiality is the most important when getting help with mental disorder. I want to keep it as secret as possible and sometimes you don’t even want the professional to know you because sometimes you feel insecure about yourself.* (#16) • *Definitely confidentiality, nobody likes the idea of others thinking they have a problem. But by this I don’t just mean private counseling but actually setting up appointments and things like that.* (#7) • *I think building the skills and confidence myself, with the help of your tools, will help me go further with gaming reduction.* (#10) • *Accountability and some form of consultation would be good. Booklets or handouts containing tips would be good, maybe something regarding logging time online, or time spent doing other stuff, and some form of online based community with others.* (#2*)* • *I’ve been trying to tackle other elements around addiction such as alcohol and marijuana but the truth is I have an addictive personality that extends to many areas of my life.* (#4) • *Someone who understands game culture. I feel like having to explain concepts and things like that all the time would make me feel like I couldn’t relate to the person on this topic.* (#7)

The *ability to seek* support was informed predominantly by beliefs that in-person treatment-seeking for problem gaming was inappropriate. There was low knowledge of service options and a perception that if help was sought, then services would not be willing or able to respond (33% of statements). Some participants perceived that the individual should be able to solve the problem themselves and that the issue was not as serious as other conditions (i.e., illicit drug use). Informing this view was social group responsivity that either minimised the problem or suggested the solution was simple (‘just stop’). Participants reported feeling embarrassed at having to seek help. They were also embarrassed to tell friends they needed to cut back on gaming for a while. There was also a perception that health resources would be made available according to the burden of disease, where gaming was less of a priority concern (due to it not being associated with serious harm). Some participants who had previous experience with addiction or health care services reported concerns on the appropriateness of service models that were either adolescent-centric (thereby being more family-centred in their approach) or focused on a disease model of addiction (abstinence over harm minimisation).

The *ability to reach* services referred to the ease of access and being located where they were most needed (22% of statements). The absence of a service system for gaming problems was highlighted in this theme, whereby participants who had sought treatment from addiction or private providers had to travel long distances to seek help or instead sought help online from international sources. They thought help should be in a convenient physical location such as co-located with university or other health care services such as those that address substance use problems (e.g., alcohol, cannabis). There was a preference for a range of different modalities for treatment which included online and in-person, as well as individual and group options. Online treatment was especially attractive for its convenience, time efficiency (access or engagement), and that as it allowed participants to type instead or talk (which was preferable initially for introverted help-seekers). There was also a view that some service options should be local, immediate, and available 24/7, which would help to address urgent issues before they became more desperate, such as the need to decrease gaming time during exams.

The *ability to pay* related to the direct costs of in-person treatment as well as other facilitating factors (10% of statements). There was a preference for direct costs to be assessed according to employment status and income. For those with low income there was an expectation of paying around NZD$20p/h for the service and upwards of NZD$100p/h for those that were employed. Factors impacting on *ability to pay* included recent transitions from home to independent living arrangements. Other factors included over-spending on gaming such as the frequent purchase of loot boxes, new games and other products such as cards and tokens.

The *ability to engage* with a service related to the fit between client need and the content, quality, and delivery modality of treatment (26% of statements). There was a view that services should be tailored according to the amount of treatment needed and the presenting problem. The range of treatment needs was extensive and included gaming-focused needs such as enhancing self-efficacy, competency and skill development in limiting or reducing gaming. Treatment needs also included co-morbidities such as addiction (cannabis and alcohol) as well as depression and anxiety. Other areas requiring assistance included sleep and stress management, social skills, and time management. The perceived quality of the treatment and relationship with the provider was perceived as important. This focused on the development of rapport through unconditional positive regard with a general empathic and non-judgemental approach. Participants also preferred treatment providers to understand gaming as well as the wider context of gaming culture (e.g., social norms).

### Service system components

**Fig. 2:**
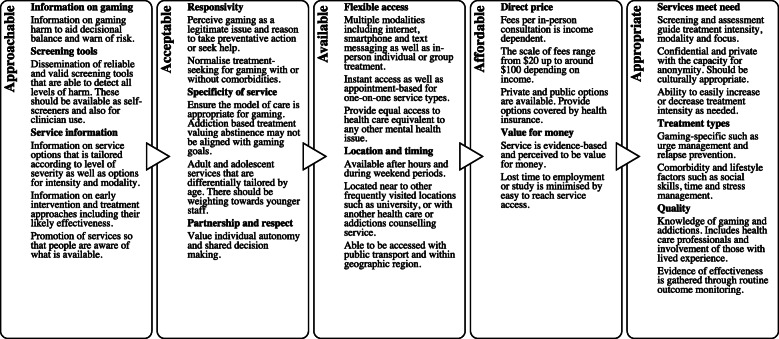
Key components of a person-centred approach to early intervention, and treatment of gaming problems

Figure 2 depicts the five components of an accessible health care system response. The *approachability* of services related to the importance of information on gaming problems, screening tools and the provision of service information. There was a need for evidence-based information on the nature and risk of gaming problems. This information would be ideally supported by valid and reliable screening tools that can be self-administered or integrated into various early intervention and treatment phases. Participants expressed confusion and uncertainty as to whether they really had a problem, indicating a need for clear feedback on problem severity and prognosis, especially for those experiencing lower levels of severity. While there are currently few community-based treatment options, participants indicated a need for clear service information that could guide decisions about service options based on level of severity (mild to severe problems) as well as modality of delivery (e.g., online, self-help, in-person).

*Acceptability* of services involved themes of responsivity and specificity, and values of partnership and mutual respect. Participants expressed a need for high quality specialist gaming services that have in-depth knowledge of gaming and its prevention and treatment. Responsive services would value taking preventive action or treatment-seeking and promote this as a normal and reasonable response to gaming issues. Gaming support, resources and services should be established for adolescent and adult gamers and avoid a ‘one-size-fits-most/all’ approach. Clinical staff should be experienced in working with young people and have relevant gaming knowledge.

The *availability* of services related to flexible access and location of the provider. Participants expressed a need for a multi-modal system that supported online, text messaging, smartphone, and in-person treatment accessible through self-help, individual and group-based formats. Participants said services should be available after-hours including evenings and weekends as well as located in or near health care centres or universities. Where travel was required, services should be able to be accessed via public transport and ideally located in each major centre. The *affordability* of in-person services was proposed to be income-based and should represent value for money. Service costs were proposed as ranging from $20 upwards depending on private or public funding.

Services should be *appropriate* to meet the needs of a potentially heterogeneous group of gamers and their family. To be *appropriate*, a person-centred approach should include valid and reliable screening tools that can inform early intervention and treatment plans and the selection of modality and intensity of treatment. This means supporting a transition between service types and intensity whereby individuals are able to alter care plans and arrangements as required (akin to a stepped care approach). Co-locating treatment within mental health services would ensure that co-morbidities are addressed, however there should also be an option whereby the treatment is primarily focused on gaming-specific issues. The workforce should have an in-depth knowledge of gaming and addictions including gaming culture (e.g., not letting your friends down) and potential comorbidities (e.g., anxiety). Participants were positive towards talking with other people with lived experience in terms of understanding how they regained control over their gaming. Identified clinical approaches included the delivery of urge management, relapse prevention and psychosocial rehabilitation via replacement activities. Services should be routinely assessed for quality and compliance with standards with these evaluations available to all clients.

## Discussion

The aim of this study was to understand the health care needs of individuals with gaming problems. We applied a person-centred framework for conceptualising the components of a support system for the identification, early intervention and treatment of gaming problems. Gamers’ perceptions and experiences reflected the components of the framework in terms of facilitators and barriers to the *ability to perceive*, *seek, reach, pay* and *engage* with treatment. This approach identified critical issues in healthcare for gaming problems that also arise across the spectrum of addictive disorders (e.g., barriers to help seeking such as shame, stigma and health beliefs around the seriousness and susceptibility of having a problem) as well as use of health care more broadly (being able to navigate, locate and reach the right type of support at the right time). The second aim was to identify the components of a healthcare system informed by the framework while accounting for participant expectations and needs. Critical discussion of this system included the need for multi-modal options that catered for mild to severe gaming problems. It also flagged the usefulness of transdiagnostic treatments that could address a range of comorbidities including mood disorders, substance use, relationship difficulties, social skills and adaptive functioning.

Gaps in health care identified in this study related to the adequacy of screening and assessment, access to evidence-based treatment and routine outcome monitoring. Screening and assessment has been identified as a major problem in gaming research that has a direct impact on service ability to provide valid and reliable feedback to gamers [24, 60]. Inconsistencies in definitions and screening are a barrier to offering the right treatment that matches client need and preferences. In terms of access to evidence-based treatment, there are very few options for those with less severe problems. Promising work is being conducted in the area of structured cognitive behavioural therapy programs [37, 61]. However, there are currently few RCTs for gaming problems suggesting that the development of an evidence-based treatment model is some way in the future [37, 61].

Some participants expressed uncertainty about the legitimacy of in-person help for gaming problems. Sixteen of twenty participants in our study met the cut-off for gaming problems inclusive of five who reported more serious problems. This meant the majority of our help-seeking participants who accessed a brief self-help intervention [53] were not experiencing severe problems which required in-person treatment. These finding are in part consistent with the broader issue of the concept of problem gaming and that few people with these issues seek in-person help [2, 11–19]. It highlights the need to develop a diverse range of early intervention and support options. Studies also indicate gamers engage with a broad range of self-managed cognitive and behavioural strategies to limit their gaming [62] and are able to adhere to personal change goals [63]. The current study highlights the need to consider a stepped care service system that is inclusive of low-severity (e.g., brief interventions such as personalised normative feedback) and low-intensity service options (e.g., self-directed treatments) as well as options that are more intensive (e.g., in-person and group treatment).

A limitation of the field is the absence of quality information on people across the continuum of severity, including case reports and clinical data. To address this problem, early intervention and treatment should include routine outcome monitoring which tracks the scope of the problem, patterns of use, and responsivity to different approaches to reducing gaming problems [64]. Ideally, routine outcome monitoring is informed by international consensus on a minimum dataset with a set of recommendations for clinical outcomes (for early intervention and treatment) and process measures. In particular, there is a need to better understand how problematic gaming may intersect with issues of comorbidity, such as mood disorders, and how such considerations may influence case formulation and treatment planning. Less is known about the efficacy of structured treatments for gaming problems, including psychological and pharmacological treatment, in the context of other mental disorders, because such issues are often excluded from trials and other intervention studies.

This is the first study to consider the optimal components of a service system for gaming problems from a holistic perspective but this work had several limitations. First, the study involved a self-selected sample of help-seeking adults who provided qualitative data. Qualitative studies are not intended to be generalisable and in the current study data were analysed to identify patterns in help-seeking preferences and experiences. These patterns were mapped onto an established model outlining the potential components of a comprehensive health care system. Future research should identify the relevance, applicability and generalisability of these findings using quantitative methodologies as well as consensus-based approaches to responding to gaming problems. Second, we believe that the model reflects the wide range of issues that need to be considered when establishing a co-ordinated clinical response to gaming problems. However, the nature of the sample means there would be additional country specific needs that should be incorporated (e.g., cultural considerations). Finally, participants in this study were motivated to change their gaming behaviour and were in the process of seeking help. Future research should examine the perspective of gamers across the continuum including those that have a problem but do not want to change as well as those who have lived experience of a range of different treatment services.

## Conclusions

A comprehensive health care approach for gaming problems is currently in its infancy and there are numerous access and delivery issues for support and access that are still to be resolved. A challenge that has received relatively less attention has been the issue of treating gaming problems and its comorbid disorders. Our findings are consistent with lessons from the gambling field that suggest treatment approaches need to be tailored according to readiness to change [65] and specific needs such as co-occurring issues. The pathways model of problem gambling outlines three different profiles of gamblers: (i) behaviourally conditioned, (ii) emotionally vulnerable, (iii) impulsive/anti-social [66]. The model recommends treatment is matched accordingly whereby those who are behaviourally conditioned may benefit from less intensive brief interventions. In comparison, those who present with comorbidities such as mood disorders would likely benefit from more intensive treatment that covers a range of different presenting issues. The proposed model in the current study is consistent with this approach in terms of recommending a range of different service options that are tailored to the needs of individuals. Currently, health care systems for gaming problems lack a stepped care approach whereby a gamer can transition between levels of treatment as needed [24].

The current study has identified issues associated with the establishment of a service system for gaming problems. A key issue with establishing a service system is the identification of an appropriate workforce who has the skills and expertise to administer the range of different service responses. Future research might identify existing capacity and how it aligns with the expertise identified in the current study.

## Data Availability

The datasets and materials used and analysed during the current study are available from the corresponding author on reasonable request.

## Abbreviations

ICD-11: International Classification of Diseases
CBT: Cognitive and Behavioural Therapy
GAS: Gaming Addiction Scale
